# Molecular and functional correction of a deep intronic splicing mutation in *CFTR* by CRISPR-Cas9 gene editing

**DOI:** 10.1016/j.omtm.2023.101140

**Published:** 2023-10-18

**Authors:** Amy J. Walker, Carina Graham, Miriam Greenwood, Maximillian Woodall, Ruhina Maeshima, Michelle O’Hara-Wright, David J. Sanz, Ileana Guerrini, Ahmad M. Aldossary, Christopher O’Callaghan, Deborah L. Baines, Patrick T. Harrison, Stephen L. Hart

**Affiliations:** 1 Genetics and Genomic Medicine Department, UCL Great Ormond Street Institute of Child Health, London, UK; 2 Institute for Infection and Immunity, St. George’s, University of London, London, UK; 3 Department of Physiology, BioSciences Institute, University College Cork, Cork, Ireland; 4 Infection, Immunity & Inflammation Department, UCL Great Ormond Street Institute of Child Health, London, UK

**Keywords:** cystic fibrosis, CRISPR-Cas9, nanoparticles, splice mutation, targeted excision

## Abstract

Cystic fibrosis (CF) is an autosomal recessive disorder caused by mutations in the *CFTR* gene. The 10th most common mutation, c.3178-2477C>T (3849+10kb C>T), involves a cryptic, intronic splice site. This mutation was corrected in CF primary cells homozygous for this mutation by delivering pairs of guide RNAs (gRNAs) with Cas9 protein in ribonucleoprotein (RNP) complexes that introduce double-strand breaks to flanking sites to excise the 3849+10kb C>T mutation, followed by DNA repair by the non-homologous end-joining pathway, which functions in all cells of the airway epithelium. RNP complexes were delivered to CF basal epithelial cell by a non-viral, receptor-targeted nanocomplex comprising a formulation of targeting peptides and lipids. Canonical *CFTR* mRNA splicing was, thus, restored leading to the restoration of CFTR protein expression with concomitant restoration of electrophysiological function in airway epithelial air-liquid interface cultures. Off-target editing was not detected by Sanger sequencing of *in silico*-selected genomic sites with the highest sequence similarities to the gRNAs, although more sensitive unbiased whole genome sequencing methods would be required for possible translational developments. This approach could potentially be used to correct aberrant splicing signals in several other CF mutations and other genetic disorders where deep-intronic mutations are pathogenic.

## Graphical abstract

**Figure:**
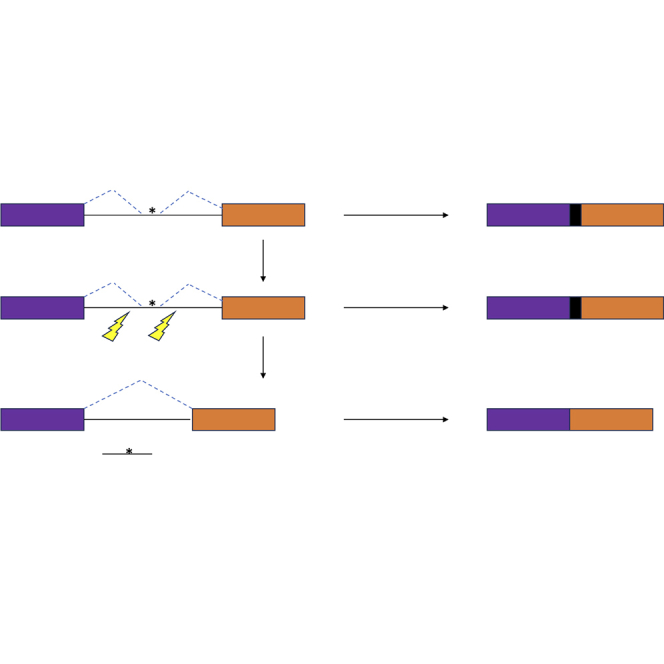

## Teaser

Hart and colleagues describe targeted excision from *CFTR* of the deep intronic *CFTR* variant 3849+10kb C>T. This variant was corrected in CF primary cells by delivering pairs of gRNAs with Cas9 protein in RNP complexes that introduce double-strand breaks to flanking sites to excise the mutation.

## Introduction

Cystic fibrosis (CF) is an autosomal recessive disease that affects around 1 in 2,500 live births in the UK.^1^ The clinical features of CF are high sodium levels in sweat, pancreatic insufficiency, biliary and gastrointestinal disease, and respiratory disease. CF is caused by mutations in the gene encoding the cystic fibrosis transmembrane conductance regulator (CFTR), an anion channel regulated by cyclic AMP-dependent phosphorylation.^2^^,^^3^^,^^4^ CFTR is crucial for trans-epithelial chloride and bicarbonate transport and is found in the secretory epithelia of many organs, including the lung, pancreas, and digestive and reproductive tracts.^4^

Over the last decade, there has been substantial progress in the treatment of CF with modulator therapies that correct defects in the CFTR protein, such as protein folding and ion channel gating properties.^5^^,^^6^^,^^7^^,^^8^^,^^9^ Highly effective triple combination drugs are now available for more than 90% of people with CF who have one or two copies of the F508del allele, leaving approximately 10% of patients with no treatment options, such as those with *CFTR* nonsense and splice site mutations. The c.3178-2477C>T (3849+10kb C>T) (cftr2.org) *CFTR* variant is the tenth most common and the focus of correction by gene editing in this study.^10^^,^^11^^,^^12^

Gene editing by CRISPR-Cas9 has opened up a wide range of new opportunities to correct CF-causing mutations including gene editing by homology directed repair (HDR), base editing, and prime editing, as reviewed recently.^13^ Each approach has benefits and shortcomings so that a range of strategies are likely to be required in seeking to treat all non-druggable *CFTR* variants. The non-homologous end-joining (NHEJ) pathway for the repair of double-strand breaks is efficient but error prone, introducing random insertions or deletions (indels) at the repair site, and so not suitable for accurate repair of mutations in the coding region of *CFTR*. However, the NHEJ pathway offers advantages in that, unlike HDR, it is effective in all cell types in the lung, independent of cell-cycle stage.^14^

The 3849+10kb C>T *CFTR* variant causes aberrant splicing of *CFTR* mRNA due to the creation of an intronic, cryptic splice signal that results in the creation of a pseudoexon containing an in-frame TAA stop codon, generating a truncated, non-functional protein.^15^ Correction of this mutation was demonstrated previously in a mini-gene assay in HEK293T cells,^16^ but these cells do not allow for the analysis of functional correction, and so we have now progressed this approach to primary cells from CF donors homozygous for the *CFTR* 3849+10kb C>T variant, and assessed the efficacy of functional correction in air-liquid interface (ALI) cultures.

**Figure 1:**
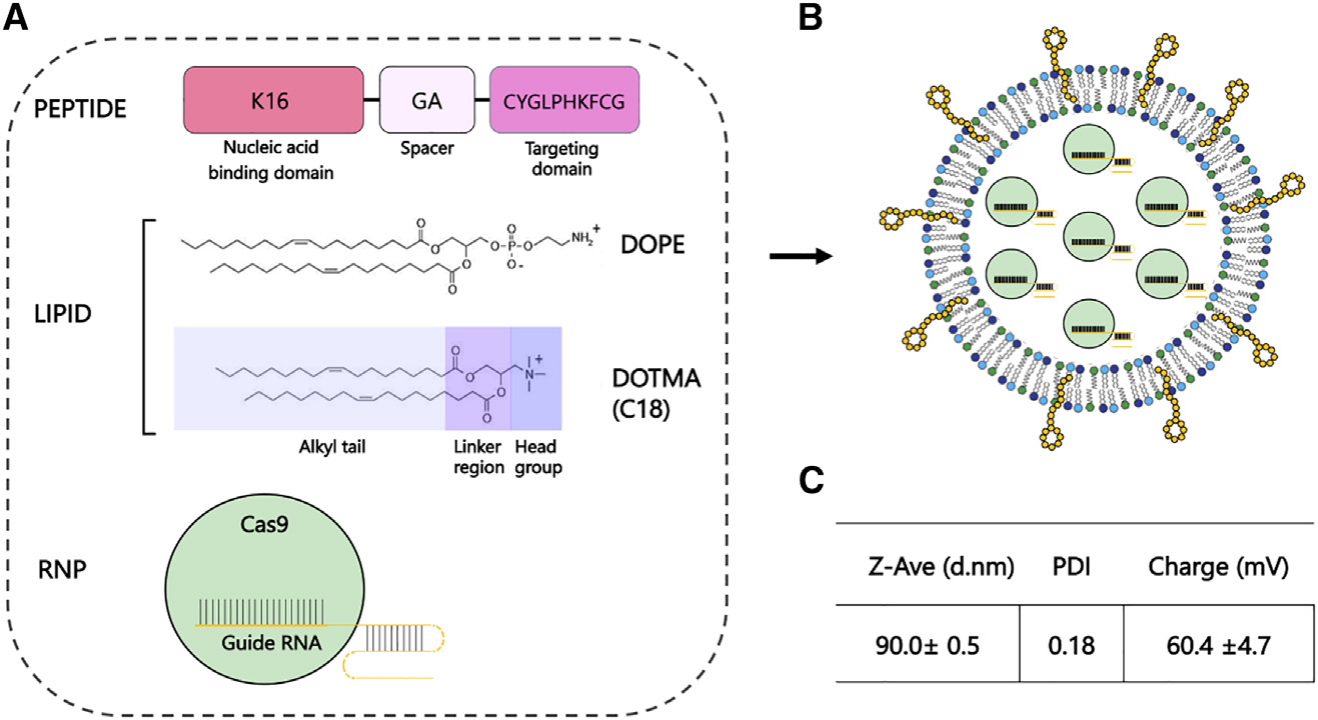
Components of the RTN (A) The targeting peptide is composed of three regions: a nucleic acid binding domain with a cationic K16 motif, a receptor-targeting ligand for binding to the cell membrane, and a short linker region to reduce steric interference. The lipid moiety is composed of a positively charged cationic lipid, DOTMA, and a neutral helper lipid, DOPE. (B) Schematic of nanocomplex. (C) Biophysical characterization of RTN for size and charge.

Basal epithelial cells have limited replication capacity and so a cell model comprising primary human bronchial epithelial cells homozygous for the 3849+10kb C>T (“CFBE3849” cells) were transduced with the gene encoding the polycomb ring finger protein, *BMI1.* BMI1 delays the onset of senescence, extending the proliferation capacity of basal cells from 2 to more than 20 passages, while retaining their differentiation capacity in ALI cultures.^17^ We have also focused on the challenge of delivery with a novel nanoparticle formulation developed in previous studies on plasmid DNA (pDNA) and small interfering RNA (siRNA)^18^^,^^19^^,^^20^^,^^21^^,^^22^^,^^23^ for RNP transfections comprising a mixture of bifunctional peptides and lipids termed a receptor-targeted nanocomplex (RTN) that enable electrostatic packaging of the RNP (Figure 1), target the nanocomplex to epithelial receptors and endosomal release of the RNP.

## Results

### Receptor-targeted nanocomplexes for ribonucleoprotein transfections

Nanocomplexes were prepared by mixing Cas9/guide RNA (gRNA) ribonucleoprotein (RNP) complexes with peptides and lipids at optimized ratios (Figure 1). The peptide features a 16-lysine, cationic binding domain, designed originally for packaging pDNA and mRNA, and a cyclic peptide motif (-CYGLPHKFC-) for cell targeting, with the two functional domains separated by a short spacer. The targeting motif was identified in earlier work by biopanning a phage display peptide library on an airway epithelial cell line.^24^ The receptor is unknown, but the peptide was found to promote targeted transfection of epithelial cells, as well as other cell types.^24^ The lipids comprise a one-to-one molar mixture of cationic 1,2-di-O-octadecenyl-3-trimethylammonium propane (DOTMA) and the fusogenic, neutral lipid 1,2-dioleoyl-sn-glycero-3-phosphoethanolamine (DOPE). The cationic mixture of peptides and lipids is very effective for nucleic acid packaging,^23^^,^^25^^,^^26^^,^^27^ but we have now shown that it is equally effective for packaging of Cas9/gRNA RNP complexes, forming discrete nanoparticles of 90 nm with a positive surface charge of approximately 60 mV. All transfections were performed with lipid/peptide packaged formulations of the RNPs unless stated otherwise.

Preliminary transfections with GFP-targeted RNP containing GFP gRNAs in normal human bronchial epithelial (NHBE) cells stably transduced with *BMI1* and GFP revealed that the RTN formulation was non-toxic at 24 h and 48 h after transfection by a ToPro3 viability assay^28^ (Table S1).

### Validation of pairs of gRNAs

HEK293T cells expressing wild-type *CFTR* were co-transfected with RNPs with dual gRNAs comprising U1 or U3 guides upstream of the mutation, and D1 on the downstream side, then the ability of the combination of gRNAs to excise the region of interest in *CFTR* intron 22 was assessed by gel analysis of PCR products spanning the target region. Single band products were detected from un-transfected cells and cells transfected with a single gRNA; however, when dual pairs of gRNAs were co-transfected, a smaller product of 141 bp (U1 D1) 179 bp (U3 D1) was visible below the parental band, which correlated with the size of the predicted deletion. The deletion efficiency was estimated by gel densitometry to be 12% and 15% for U1 D1 and U3 D1 pairs, respectively (Figures S1A and S1B), confirming the potential for targeted excision of the 3849+10kb C>T intronic mutation.

### Validation of gRNAs in CFBE3849 cells

**Figure 2:**
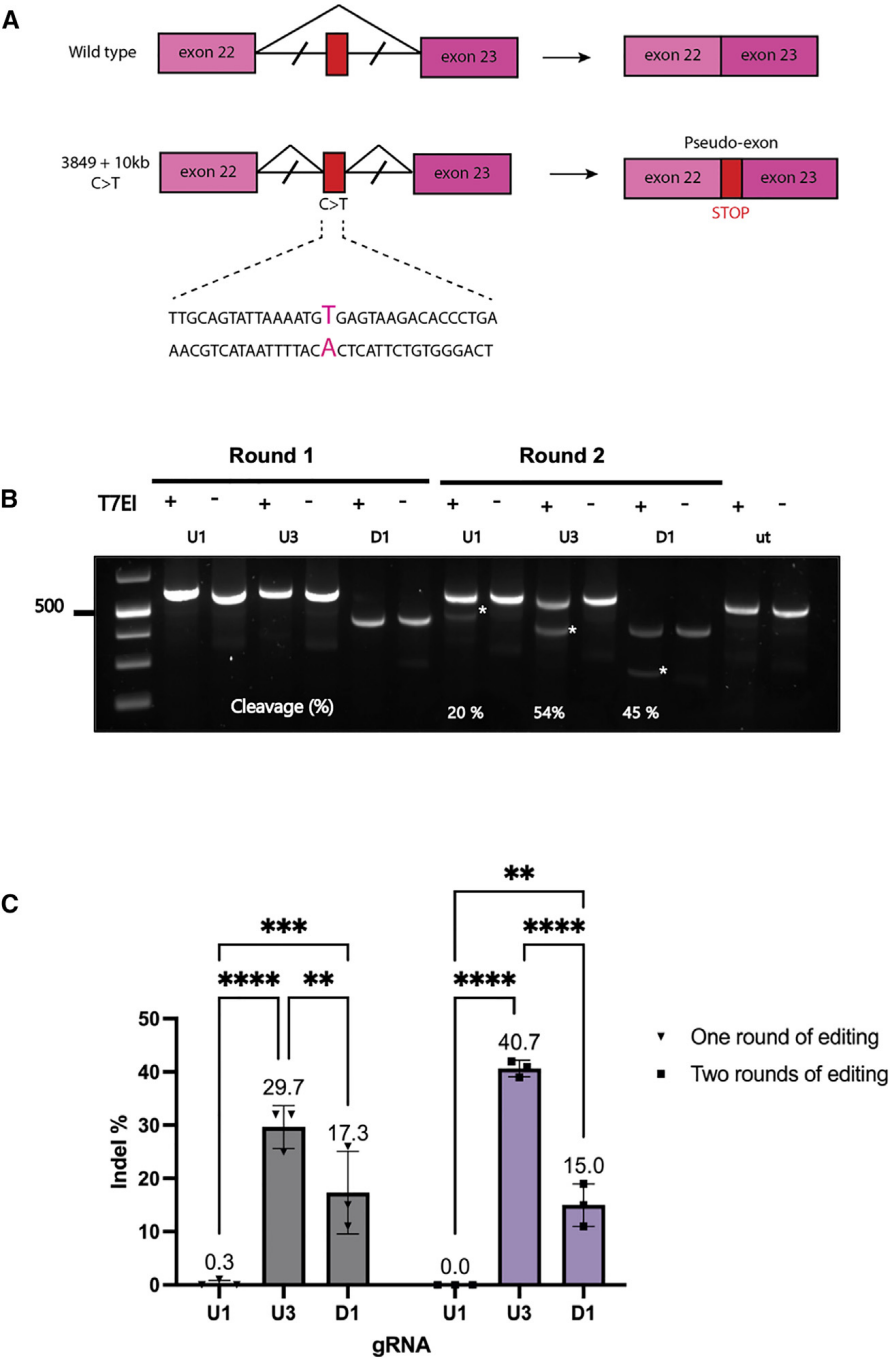
gRNA validation in CFBE3849 cells (A) Schematic of CFTR WT (top) and 3849+10kb C>T mutation (bottom) exons 22–23. Exons are shown as boxes, and introns as lines above the nucleotide sequence surrounding the CFTR 3849+10kb C>T mutation (highlighted in pink) with the different splice products shown on the right. This mutation leads to the incorporation of an 84-bp pseudo-exon containing a STOP codon (red). (B) CFTR intron 22 was PCR amplified with oligonucleotides CFTRex22_F and CFTRex22_R then analyzed by T7EI mismatch assay. Agarose gel shows products from CFTR intron 22 in CFBE3849 cells transfected with individual gRNA RNPs. Cleaved product bands are indicated with an asterisk and estimated by densitometry. (C) Editing efficiency of individual gRNAs as assessed by ICE analysis of PCR sequenced fragments after one or two rounds of transfection with RNPs with individual gRNAs. Statistical analysis by two-way ANOVA with Tukey’s multiple comparison test. Significance of ∗∗p < 0.01, ∗∗∗p < 0.001, ∗∗∗∗p < 0.0001. n = 3.

We next evaluated the efficacy of the strategy of targeted excision in *CFTR* intron 22 in CF primary bronchial epithelial cells, homozygous for the 3849+10kb C>T variant, transduced with *BMI1* to enable long-term maintenance and differentiation^17^ (Figure 2A). Three gRNAs that were described previously in a minigene editing study for the same variant were selected for screening for continuity with the previous study.^16^ The indel frequency from each gRNA was first evaluated in CFBE3849 basal epithelial cells. Cells were transfected with RNP lipid-peptide nanocomplexes containing single gRNAs and Cas9 nuclease; then, 48 h later, genomic DNA was extracted for T7 endonuclease I (T7EI) analysis or transfection was repeated.

After two rounds of transfection, T7EI gel analysis revealed cleavage products of the expected size (Figure 2B). All three gRNAs created indels within *CFTR* intron 22, the most efficient being U3 and D1, with 54% and 45% indel frequency, respectively. Analysis of PCR-amplified DNA from *CFTR* intron 22 by Tracking of Indels by Decomposition (TIDE) showed indel frequencies for U3 and D1 gRNAs of 55.9% and 37.5%; U1 was the lowest at 8% (Figure 2C). The indel spectrum of each of the gRNAs showed that 43% of indels from U3 RNP treated cells involved a 1-bp deletion (Figure 2E), while the most common indel in D1-treated cells was a 1-bp insertion (Figure 2F). The indel frequency was extremely low with U1-treated cells (Figure 2D) and so U3 and D1 guide pairs were used in all subsequent transfections.

### Targeted excision of *CFTR*3849+10kb C>T in CFBE cells

**Figure 3:**
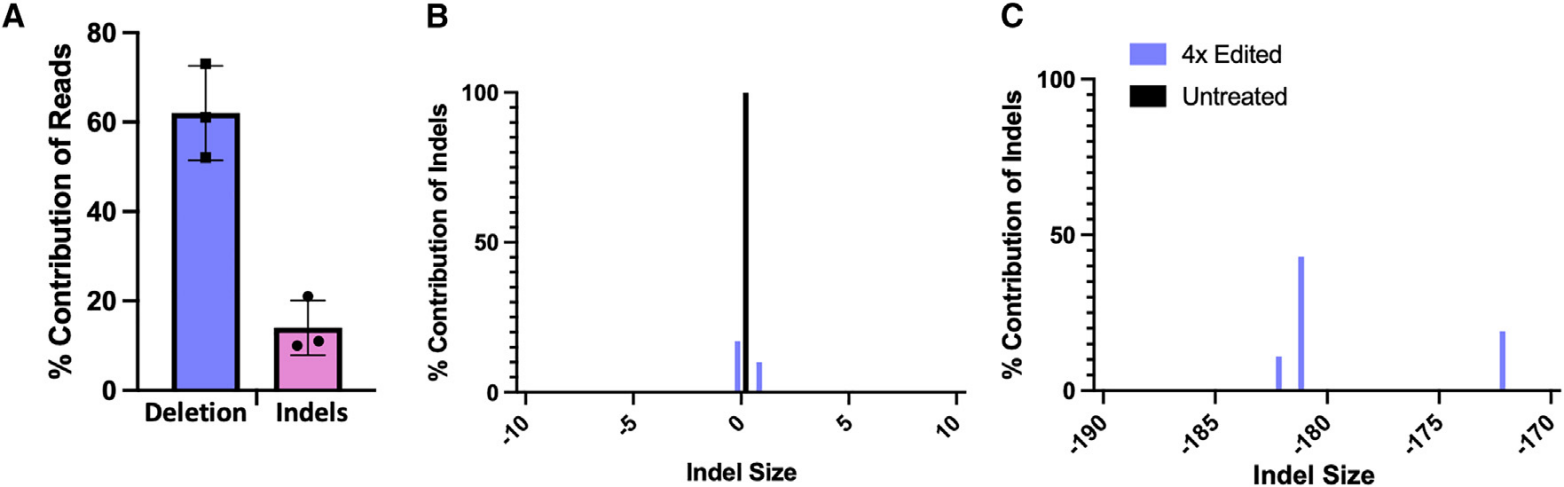
Targeted excision of the deep intronic mutation (A) CFBE3849 cells were transfected four times at 48-h intervals with Cas9 RNPs with U3D1 gRNAs then PCR DNA sequences analyzed for indels in CFTR intron 22 by ICE. Representative ICE analyses of individual samples for, (B) indels, and, (C) larger, targeted excision events.

CFBE3849 cells were co-transfected with Cas9 plus U3 and D1 gRNAs, with RNPs in lipid-peptide nanocomplexes, to create a targeted excision. Editing efficiency was assessed using Inference of CRISPR Edits (ICE) DNA sequence analysis software, which, is more suitable for detecting larger genomic deletions than TIDE. Approximately 62% of sequences displayed the ∼178-bp-targeted excision (Figure 3A) with an additional indel frequency of 14% at the U3 and D1 sites. Representative Sanger sequence traces from edited cells are shown in Figure S2.

### Off-target indel analysis

The gRNAs used in this paper were the same as in a previous paper in a minigene model, as this study is a follow-up, evaluating editing in primary cells with the same variant.^16^ Edited CFBE3849 cells were analyzed for indels at potential off-target sites (Tables 1 and 2). The top five sites with the closest similarity to each gRNA identified by CRISPR-Cas9 Target Online Predictor (CCTOP) all had four mismatches from the target in non-coding intergenic or intronic regions except one other intronic site, with three mismatches from the D1 gRNA, that failed to amplify by PCR despite repeated attempts. PCR amplification and Sanger sequencing at each of these sites failed to detect indels while a high efficiency of indels was observed for each on-target site in intron 22. Further *in silico* screening of guides U3 and D1 was also performed by CRISPOR (Datas S2 and S3) which factors in experimental data, and COSMID (Datas S4 and S5), which considers small bulges in the target DNA or gRNA due to indels of the target site. CRISPOR analysis revealed 103 target sites for D1 and 179 sites for U3 with CDF values of greater than 0.2, therefore with the potential for off target double-strand breaks. Further analysis by unbiased, whole-genome screening approaches such as CIRCLE-Seq^29^ or GUIDE- Seq^30^^,^^31^ would be required for deeper analysis of off-target editing sites.

**Table 1:** Details of predicted off-target sites from CCTOP

Site	Genomic location	Strand	Mismatches from target	Guide sequence	Feature	% indel formation
**D1 target site**	chr7:117280121-117280143	+	N/A	TTGATCCAACATTCTCAGGG	intronic	83
D1 Off- target site 1	chr15:89498076-89498098	+	4	GGGATCTCACATTCTCAGGG	intergenic	0
D1 Off-target site 2	chr12:28215823-28215845	–	4	TTAAGGTAACATTCTCAGGG	intergenic	0
D1 Off- target site 3	chr1:90843519-90843541	+	4	TTAGTTCATCATTCTCAGGG	intergenic	0
D1 Off- target site 4	chrX:152104444-152104466	–	4	CTTACCCAACCTTCTCAGGG	intronic	0
D1 Off- target site 5	chr6:14178697-14178719	+	4	TGGATGCTGCATTCTCAGGG	intergenic	0
**U3 target site**	chr7:117279942-117279964	+	N/A	CTTGATTTCTGGAGACCACA	intronic	83
U3 Off- target site 1	chr12:106229757-106229779	–	4	AATGTTTACTGGAGACCACA	intergenic	0
U3 Off- target site 2	chr8:21719671-21719693	+	4	TTTACTTTGTGGAGACCACA	intergenic	0
U3 Off- target site 3	chr12:130167000-130167022	–	4	TTTGCCTGCTGGAGACCACA	intronic	0
U3 Off- target site 4	chr1:112030339-112030361	–	4	GTTTATATTTGGAGACCACA	intronic	0
U3 Off- target site 5	chr3:23175060-23175082	+	4	CCAGATTACAGGAGACCACA	intergenic	0

D1 and U3 gRNAs are highlighted. For the off-target sites, mismatches to the on-target spacer sequences are marked in bold. Indel formation is reported as a percentage of Sanger sequence reads that contained indels after treatment with both gRNAs.

**Table 2:** Primer sequencers used for PCR sequencing in off target analysis

Gene ID	Primers
RP11-63A23.2	D1 OTE 1 Fwd: GACCCAGGAGTAAGCACTCACAA D1 OTE 1 Rev: CTTTCTCTGCACCCTCTATAAGAGC
CCDC91	D1 OTE 2 Fwd: GGATACGTCAAGCCTAATGAGAGT D1 OTE 2 Rev: AAGACCTTGAGGGAGGGAGAAATTG
NA	D1 OTE 3 Fwd: CCTGAGCAAGCCTTAGTGGTTC D1 OTE 3 Rev: TCATGTGAGAGAGAGCCTGAGTTAG
ZNF185	D1 OTE 4 Fwd: CAACTGGCATAAAGAGGTCTGGG D1 OTE 4 Rev: TCATGGCTTTGCTATCTCCCAG
RNU7-133P	D1 OTE 5 Fwd: CAGAGCAGACTACGTGCTTACA D1 5 Rev: CTGAAGCGTGGAGAAGTGAAGG
CASC18	U3 OTE 1 Fwd: GCAGTGTGATGAACGTGGTGA U3 OTE 2 Rev: GGAGGATGTGACAGATTGATTGCA
DOK2	U3 OTE 2 Fwd: CCACCAAGCCCAGCAGATTT U3 OTE 2 Rev: GAAAGGAAGGAAGATGAGCAGTGG

### Splicing correction in primary airway cells

**Figure 4:**
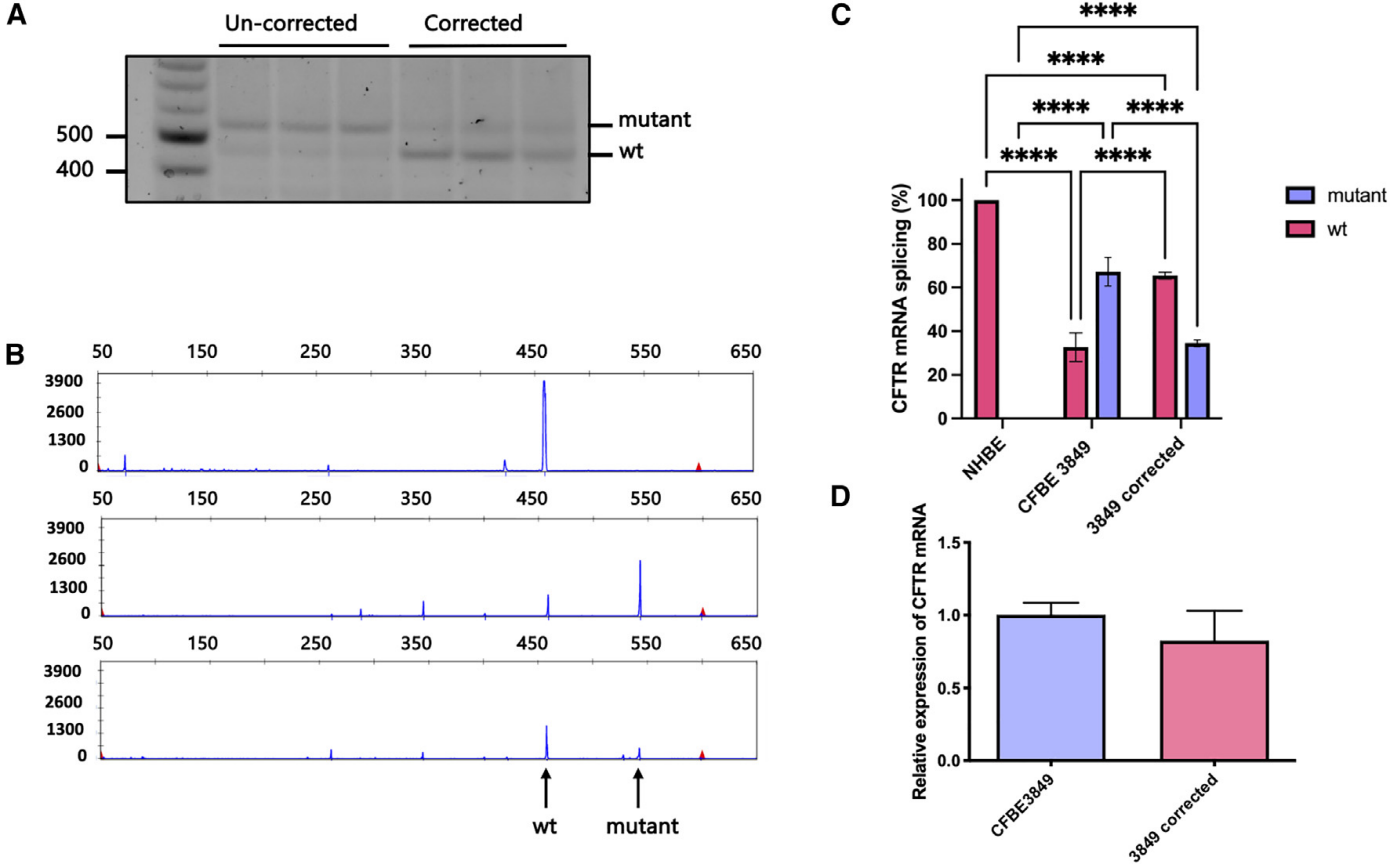
CFTR mRNA splicing analysis in CFBE3849 cells (A) Agarose gel electrophoresis analysis of RT-PCR products from edited and non-edited CFBE3849 cells. The top band indicates the CFTR transcript containing the pseudoexon, while the bottom band is WT CFTR. (B) Representative electropherogram analysis of RT-PCR products of WT NHBE (top), CFBE3849 (middle), and 4× edited CFBE3849 cells (bottom). Wild-type and mutant CFTR transcripts are indicated with arrows. The x axis indicates fluorescence intensity and the y axis the fragment size in number of bases. (C) Quantification of CFTR mRNA splicing in NHBE and corrected and non-corrected CFBE3849 cells calculated from the area under the curve for the WT fragment relative to the mutant in the electropherogram (n = 3). (D) qRT-PCR data of CFTR mRNA of corrected and un-corrected cells normalized to β-actin transcripts. Results are presented as mean ± SD. Statistical analysis by two-way ANOVA with Tukey’s multiple comparison test. ∗∗∗∗p < 0.0001.

NHBE, un-edited CFBE3849 cells, and 4× edited CFBE3849 cells were seeded onto semi-permeable membranes and maintained in ALI culture until differentiated as shown by the TEER value (>300 Ω), and the appearance of motile cilia and mucus, typically after 3 weeks of ALI culture. RNA was then extracted from the cells, reverse-transcribed, and amplified by PCR using a FAM-labelled forward primer, which spans the junction of exons 21 and 22, and an unlabeled reverse primer. The PCR product was analyzed by agarose gel electrophoresis (Figure 4A), then subjected to electropherogram fragment length analysis (Eurofins). NHBE cells showed 100% full-length *CFTR* transcripts, while in CFBE3849 cells 25% of transcripts were full length (wild type) and 75% contained the mis-spliced mRNA (Figure 4B). The results show that homozygous CFBE3849 cells, displayed “leaky,” residual production of correctly spliced *CFTR* mRNA, as was reported previously for people with this variant.^32^ Analysis of transcripts from cells in which the *CFTR* intron 22 splice mutation was excised showed an increase from 25% to 66% of wild-type (WT) *CFTR* transcripts (Figure 4C). Analysis of total *CFTR* mRNA qRT-PCR analysis showed no significant change in the overall level of *CFTR* mRNA between edited and un-edited CFBE3849 cells (Figure 4D), which was unexpected; we predicted the mutant transcript would be subjected to nonsense-mediated decay through nonsense mutations in the pseudoexon incorporated into the mRNA.

### Immunofluorescent analysis of edited CFBE3849 cells

**Figure 5:**
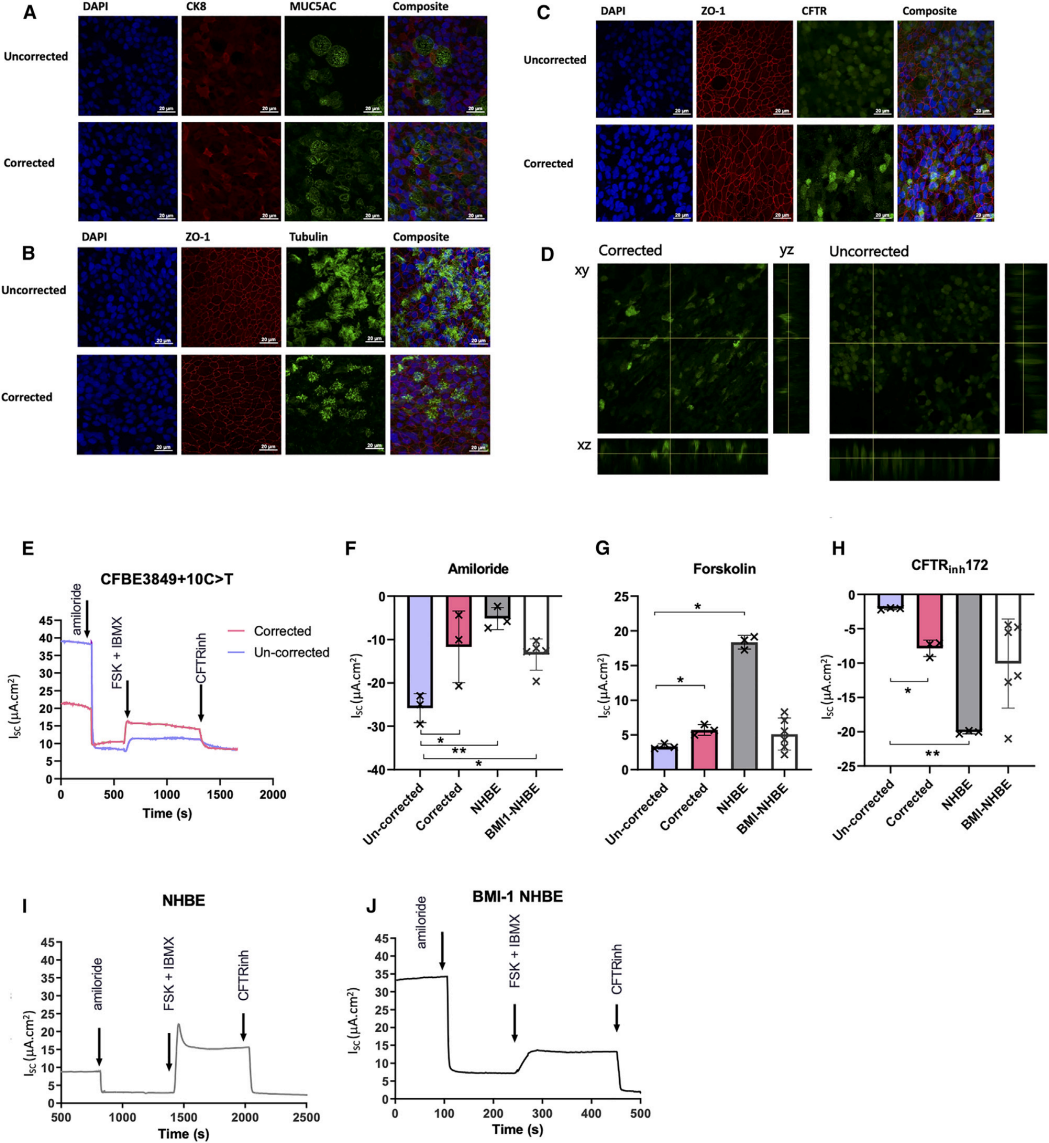
Immunofluorescence and electrophysiology analysis of edited CFBE3849 cells Immunofluorescence analysis was performed on edited CFBE3849 cells in ALI culture (A-). Merged composite images are shown in the far-right column of each panel. (A) Cells were stained for 4′,6-diamidino-2-phenylindole (DAPI) (blue), CK8 (red), and MUC5AC (green). (B) Cells were stained for DAPI (blue), ZO-1 (red), and tubulin (green). (C) Cells were stained for DAPI (blue), ZO-1 (red), and CFTR (green). (D) XY, XZ, and YZ cross-sections from segmented image stack of 3849 cells stained with CFTR. Electrophysiology studies were performed in 4× transfected CFBE3849 cells. (E) The I_sc_ traces of non-corrected and corrected CFBE3849 cells and their response to amiloride, forskolin, and IBMX, followed by CFTR inhibitor-172 as shown recorded using the Ussing technique. The ΔI_sc_ in uncorrected and corrected CFBE3849 cells, BMI1-NHBE and primary NHBE after the addition of (F) amiloride, (G) forskolin and IBMX, and (H) CFTRinh172. Positive values represent stimulation and negative values represent inhibition of Isc. The short-circuit current (I_sc_) traces of, (I) primary NHBE cells, and (J) BMI1-NHBE cells. Results are presented as mean ± SD, n = 3–6, and significant differences indicated as ∗p ≤ 0.05, ∗∗p < 0.01.

ALI cultures of CFBE3849 cells, were assessed for their expression of characteristic epithelial markers by immunofluorescent staining to validate the model. Both ALI cultures before and after editing, expressed MUC5AC, characteristic of mucin-secreting goblet cells, cytokeratin CK8, acetylated α-tubulin, the marker for cilia (Figures 5A and 5B), and zonula occludens (ZO)-1, typically found at the apical junctional complex of an intact epithelium. Thus, ALI cultures of *BMI1*-transduced CFBE3849 cells displayed many of the characteristics of a polarized epithelial culture, with distinct apical and basolateral membranes (Figure 5B).

After CRISPR-Cas9 gene editing, more intense CFTR staining was observed in edited cultures than in un-edited CFBE3849 cells, with enhanced localization to the apical membrane (Figures 5C and 5D). The residual presence of CFTR in unedited cells was expected from *CFTR* mRNA splice analysis, which suggested that CFBE3849 cells displayed background levels of canonical splicing (Figure 4C).

### Functional restoration of CFTR

Targeted excision of the splice mutation in intron 22 of CFBE3849 cells increased the level of canonical splicing and CFTR protein production, leading to the next question of whether this protein was functional as an anion channel. Electrophysiological properties of ALI cultures of edited and unedited CFBE3849 cells, and *BMI1*-transduced NHBE and primary NHBE cells were compared. Inhibition of short circuit current (I_sc_) with amiloride, reflecting activity of the epithelial Na^+^ channel (ENaC), was greater in un-edited CFBE3849 than in edited CFBE3849 cells. The change in I_sc_ (ΔI_sc_) with amiloride was like that observed in both *BMI1*-transduced NHBE (*BMI1*-NHBE) (n= 6) and primary NHBE cells (n = 3) (Figures 5E and 5F).

Activation of CFTR by forskolin and isobutylmethylxanthine (IBMX) induced a significantly greater ΔI_sc_ in edited than in un-edited CFBE3849 cells (5.7 μA/cm^2^ and 3.3 μA/cm^2^, respectively; p ≤ 0.05; n = 3). The forskolin/IBMX-generated ΔI_sc_ was similar in corrected CFBE3849 and *BMI1* NHBE cells, but lower than that of primary NHBE cells (p < 0.05, n = 3) (Figures 5E and 5G). Inhibition of CFTR with CFTR inhibitor 172 (CFTR_inh_172) produced a ∼4-fold greater decrease of I_sc_ in edited cells compared with un-edited controls (ΔI_sc_ −7.8 μA/cm^2^ and –2.1 μA/cm^2^, respectively; p ≤ 0.05, n = 3). CFTR_inh_172 ΔI_sc_ was like *BMI1-*NHBE cells but lower than that of primary HBEC (Figures 5E and 5H). Interestingly, treatment with CFTR_inh_172 inhibited all forskolin/IBMX-induced I_sc_ of edited and un-edited CFBE3849 cells and primary NHBE cells, but inhibited more than the forskolin/IBMX induced I_sc_ in *BMI1*-NHBE, indicating that there was some already activated CFTR in these cells (n = 6) (Figures 5E, 5I, and 5J). Differences between NHBE and *BMI1*-NHBE cells may be due to differences in the donor, culture differences, or the BMI1 transduction itself. Nevertheless, these data indicate functional restoration of CFTR in edited CFBE3849 cells together with concomitant lowering of ENaC activity. The increase in CFTR-dependent I_sc_ also correlated with the increased abundance of CFTR observed in edited cells (Figures 5C and 5D).

## Discussion

This paper describes a potential CRISPR-mediated therapy for *CFTR* 3849+10kb C>T, a specific CF-causing variant of *CFTR*. This mutation is the 10th most frequent *CFTR* variant (cftr2.org). Gene therapy for CF targeted to the airway epithelium has been investigated for almost 3 decades, with 26 clinical trials so far^33^^,^^34^^,^^35^^,^^36^^,^^37^^,^^38^^,^^39^^,^^40^^,^^41^ and no clinically effective therapy to date. Two of the major limitations of gene therapy by non-viral, liposomal delivery of *CFTR* cDNA include low levels of gene transfer and short-term persistence of transgene expression. Gene editing offers possible strategies to help overcome these limitations of earlier gene therapy studies for CF.

CRISPR-Cas9 genome editing technology offers precise engineering of the genome with much greater ease and a lower cost than previous gene editing techniques such as TALENs and ZFNs.^42^ As a monogenic disease, CF is an ideal candidate for CRISPR-Cas9 gene correction, and several approaches are under investigation.^14^^,^^43^^,^^44^^,^^45^^,^^46^ HDR-mediated approaches displayed relatively low levels of editing and are limited to dividing cells, thus excluding most airway epithelial cells. HDR efficiency is also limited by the need for a DNA donor template with nuclear uptake across the nuclear envelope, a major barrier to efficiency. High-efficiency repair of the ΔF508 mutation by HDR was reported in primary CF basal epithelial cells *in vitro* by electroporation with liposomal Cas9 and sgRNA, while the DNA template was delivered by AAV6, with a repair efficiency of approximately 40%, but also an indel frequency of approximately 38% in the non-corrected allele, most of which would generate *CFTR*-inactivating frameshifts and deletions.^46^ However, utilization of NHEJ-mediated repair pathway, for selective targeting of deep intronic *CFTR* mutations, offers the potential for donor-free repair in all cells carrying such mutations, including post-mitotic cells.

The *CFTR* 3849+10kb C>T variant leads to the insertion of a pseudoexon into the transcribed mRNA, leading to nonsense-mediated decay or production by generation of in-frame nonsense mutations or a non-functional protein.^47^ Targeted excision of this mutation by CRISPR-Cas9 with flanking gRNAs followed by NHEJ repair was shown to restore the transcription of canonical *CFTR* mRNA with high efficiency in a minigene plasmid model, and so the aim of this study was to investigate the therapeutic potential of this repair strategy in primary human CF cells and to evaluate a novel method of delivery of the Cas9/gRNA RNP with a RTN comprising a formulation of targeting peptides and lipids^20^ that we have shown here self-assemble into nanocomplexes of less than 100 nm with the RNP. Targeted excision in CFBE3849 cells was observed in approximately 62% of alleles with further indel frequency of 14%, which were mostly +1 indels, with the remaining reads remaining as unaltered WT sequences, after four rounds of transfection. These results demonstrated the potential of this approach to achieve the recommended therapeutic levels of *CFTR* editing, which is estimated at higher than 25%.^48^ Sanger sequencing of PCR products from potential off-target sites with the closest match for each gRNA did not detect, within the limits of sensitivity of this approach, any indels, although more sensitive, unbiased whole genome screening methods, such as GUIDE-Seq or CIRCLE-Seq, may reveal off-target events and would be required for translational development. Canonical mRNA splicing was at least partially restored by fragment length analysis, leading to increased levels of CFTR protein by immunofluorescent microscopy.

Functional testing of the corrected protein was assessed by Ussing chamber analysis of ALI cultures prepared from edited cells also indicated at least a partial improvement in ion transport activity. Restored channel activity in the corrected CFBE3849 cells was like levels in NHBE-*BMI1* cells at passage 11. Corrected activity levels were lower at 40%, compared with non-*BMI1* transduced primary NHBE cells at passage 2, suggesting lower CFTR activity after the BMI1 transduction process, although even at the lower level, 40% correction should be sufficient to restore mucociliary transport and ameliorate disease progression.^48^ In the above experiments, transfections were performed in CFBE3849 basal cells prior to establishing ALI cultures, then functional testing performed in the differentiated epithelium once differentiated after about 4 weeks. Editing of basal cells *in situ*, in the pseudostratified epithelium in ALI cultures is challenging because of the copious amounts of mucus produced in these cultures and the location of the basal cells. It was shown that accessing the basal cells by viral vectors was enhanced by pretreatment by chemical or physical perturbation of the epithelium^49^ and so this may also be possible for non-viral nanoparticles. Alternatively, accessing the basal cells may be enhanced by systemic delivery.,^50^^,^^51^ but this strategy presents its own unique technical challenges in traversing the intervening cellular layers before accessing basal cells, which await further technological developments of LNPs.

The variant pseudoexon in CFBE3849 cells encodes a downstream in-frame nonsense codon which could potentially result in nonsense-mediated decay (NMD) of mRNA. However, qRT-PCR data from CFBE3849 cells suggests there was no change in *CFTR* transcribed mRNA levels in edited cell populations, and so editing probably does not reduce NMD in contributing to the efficacy of the treatment.

All RNP transfections were performed with a non-viral, receptor-targeted nanocomplex (RTN) comprising a formulation of DOTMA/DOPE liposomes that facilitate membrane transport, particularly in escaping the endosome, and a cationic, epithelial receptor-targeting peptide that facilitates both RNP packaging and receptor-mediated uptake. The targeting peptide used in this study, YGLPHKF, was identified by phage peptide library biopanning of epithelial cells.^24^

We have previously reported the use of similar lipid-peptide nanoformulations to deliver pDNA and siRNA, to a variety of cell types and tissues, including *in vivo* delivery to murine lungs^18^^,^^19^^,^^20^^,^^21^^,^^22^^,^^23^ and now report their use for RNP delivery. The complex of lipids, peptides, and RNP forms homogeneous nanoparticles of about 90 nm in size with a cationic zeta potential, or surface charge. The nanoparticle most likely self-assembles through electrostatic interactions between the cationic peptide and liposome, and the anionic residues of Cas9 and the gRNA.^27^ During transfection, like previously described nucleic acid transfection mechanisms, epithelial receptor-mediated uptake of the RNP nanoformulation is mediated through the epithelial-specific peptide ligand,^24^ as well as the non-specific, cationic properties of the nanoparticle, followed by endosomal release mediated by the fusogenic lipid, DOPE, as previously described for pDNA delivery.^25^ The RNP is then released into the cytoplasm and transported to the nucleus by the nuclear localization peptide sequences of Cas9.

RNP delivery offer benefits of high editing efficiency, which is more transient than Cas9 delivered by viral vectors, pDNA, or mRNA, and so minimizes exposure of the host genome to the nuclease and the risk of off-target, double-strand breaks in the DNA.^52^ Packaging the RNP in a nanoparticle may also help to decrease exposure to an immune response to Cas9 *in vivo*, where innate and adaptive immune response have been reported.^53^

Recently, an alternative editing strategy to correcting the 3849+10kb C>T mutation was reported, as well as an additional splice mutation, 3272–26 A>G (also known as c.3140-26A>G) based on allele-specific editing.^44^ AsCas12a and a single gRNA were delivered via lentiviral transduction and were able to discriminate between WT and mutant sequences by removing essential splicing regulatory elements. CFTR functionality was confirmed by the swelling assay gut organoids. In addition, a CRISPR adenosine base editing strategy for repair of the 3849+10kb C>T mutation was reported, which has the advantage of avoiding the need for double-strand breaks although off-target base editing remains a risk.^54^ The NHEJ pathway has also been used to prevent NMD of *CFTR* transcripts containing the second most common nonsense variant mutation, W1282X, by deleting the region downstream of the premature stop codon.^55^ However, this approach could only be used for repair of nonsense mutations toward the 3′ end of the gene where a truncated protein may retain partial functionality, such as reported in the dystrophin gene underpinning Duchenne’s muscular dystrophy.^56^ In addition, other competing strategies include *CFTR* mRNA^57^ or *CFTR* gene replacement therapies,^58^ which can both correct, not only this mutation, but any other *CFTR* mutation. However, surface epithelial cells targeted by NHEJ repair strategies are more readily accessible to nebulized delivery than basal cells. While not as long lived as basal cells, surface epithelial cells are long lived, lasting weeks to months, and so correction of this mutation in these cells by CRISPR gene editing has the potential for more long-term benefit than mRNA, which will require redosing at intervals of 1–3 weeks. In addition, gene editing restores expression in the correct cells that are regulated by native processes, whereas mRNA or gene replacement therapies are likely to be expressed in all airway epithelial cells, with unknown consequences.

In addition to the 3849+10kb C>T mutation, there are several other CF-causing mutations to which this editing strategy could be applied. For example, 1811+1.6kb A>G (c.1679+1634 A>G) creates a very strong splice donor site in intron 12 of *CFTR* leading to 99% of transcripts containing a 49-bp pseudoexon with an in-frame TAA stop codon causing premature termination of the CFTR protein.^59^ In addition, the 3272-26 A>G variant creates a splice acceptor site 26 bp upstream of exon 20, extending the exon by 25 bp. The resultant frameshift leads to premature termination of CFTR at a TGA stop codon.^60^ Additionally, 1787+18kb A>G (c.1584+18672A>G),^61^ 1811+1643G>T (c.1680-877G>T),^62^ and 3849+40 A>G (c.3717+40A>G)^62^ could also be amenable to a targeted excision strategy. Together, these six mutations represent approximately 1.6% of individuals with CF. Furthermore, 75 other genetic disorders have been identified where deep-intronic mutations are disease causing,^63^ including monogenic diseases such as β-thalassemia,^64^ amyotrophic lateral sclerosis 1,^65^ and Leber’s congenital amaurosis 10,^66^ for which a clinical trial of targeted excision using Cas9 and two gRNAs is currently in progress (NCT03872479).^67^ Other diseases of the eye could be responsive to a targeted excision strategy include gyrate atrophy of choroid and retina and retinitis pigmentosa 11.^65^

In conclusion, we have provided evidence for targeted excision of a deep intronic *CFTR* splice mutation using the CRISPR-Cas9 system. The high efficiency and simplicity of the targeted excision approach and the potential for NHEJ repair in a wide range of cells could form the basis of a potential therapeutic intervention for CF and other diseases.

## Materials and methods

### gRNA design

Putative gRNA sequences were designed by the CRISPR design tool (http://crispr.mit.edu/) to target a region of approximately 300 bp surrounding the 3849+10kb C>T mutation, as described^16^ (Table S2). Double gRNAs were ordered as CRISPR-Cas9 crRNA and tracrRNA (Alt-R, IDT Technologies, Leuven, Belgium) and assembled in a duplex annealing buffer (30 mM HEPES, pH 7.5; 100 mM potassium acetate) at an equimolar concentration of 30 μM and incubated at 95°C for 5 min before cooling slowly to room temperature.

### Cell culture

HEK293T cells (ATCC CRL-1573; Manassas, VA), a widely used kidney epithelial cell line, were cultured in DMEM (Life Technologies, Paisley, UK) supplemented with 10% (vol/vol) fetal bovine serum (Life Technologies). Primary airway bronchial epithelial cells from a CF patient homozygous for the 3849+10kb C>T mutation (“CFBE3849” cells), were provided by Dr. Scott H. Randell (Marsico Lung Institute, Tissue Procurement and Cell Culture Core, The University of North Carolina at Chapel Hill, NC),^68^ under protocol #03-1396 approved by the University of North Carolina at Chapel Hill Biomedical Institutional Review Board. CFBE3849 cells were cultured in PneumaCult-Ex Medium (Stem Cell Technologies, Cambridge, UK) in collagen coated flasks (Sigma-Aldrich, Gillingham, UK) and were transduced at passage 2 with a lentiviral vector containing full-length human *BMI1*, as described previously,^17^ at a multiplicity of infection of 1.

### ALI cultures

Primary airway bronchial epithelial cells were seeded in collagen-coated Snap-well plates (Corning, Amsterdam, the Netherlands) at a density of 0.5 × 10^6^ per 1.2 cm^2^ in 250 μL PneumaCult-Ex Medium while 1 mL per well of PneumaCult-Ex Medium was added to the basolateral side of the membrane. Media was aspirated 72 h later from both the apical and basolateral sides of the membrane and replaced on the basolateral side with 1 mL PneumaCult-ALI Medium media. Media was then replaced on the basolateral side every 48 h until the experiments were performed in a differentiated culture at about 21 days.

### Liposome formulation and preparation of RTN

Liposomes were formed using a NanoAssemblr (Precision Nanosystems, Vancouver, BC, Canada). The cationic lipid DOTMA (C18) and DOPE (both Avanti Polar Lipids, Alabaster, AL) were mixed in ethanol at a molar ratio of 1:1 and injected into the microfluidic mixing cartridge at a flow rate of 12 mL min^−1^. The newly formed liposomes were then dialyzed overnight in SnakeSkin Dialysis Tubing (ThermoFisher Scientific, Paisley, UK) (10K MWCO, 22 mm) in 2 L sterile MilliQ water at room temperature with stirring, to remove residual ethanol. Liposomes were then sonicated in a water bath for 20 min to reduce the size, producing small, unilamellar vesicles. Liposomes were diluted to 1 mg/mL and stored at 4°C. RNP complexes were formed with gRNA and Truecut Cas9 protein v2 (ThermoFisher Scientific) at a weight ratio of 1:4 gRNA:Cas9 in OptiMEM (Life Technologies) and incubated for 5 min for the complex to form.

RNP-liposome-peptide nanocomplexes were prepared at a weight ratio of 1 (RNP):3 (lipid):4 (peptide Y; K_16_GACYGLPHKFCG) (AMSBio, Abingdon, UK) by first mixing the liposome (1 mg/mL in water) with the preformed RNP complexes (1 mg/mL), followed by the addition of peptide (1 mg/mL) with rapid mixing. The mixture was incubated at room temperature for 30 min to allow complex formation, before addition of OptiMEM to give a final RNP concentration of 6 mM.

For biophysical characterization experiments, the nanocomplex was prepared in water with 1–2 μg RNP, then incubated for 30 min at room temperature. The sample was then diluted to a final volume of 1 mL and transferred to a cuvette where size and charge (ζ potential) of the nanocomplexes were measured using a Nano ZS Zetasizer (Malvern, UK).

### Transfections

HEK293T cells were seeded in 24-well plates (Thermo Fisher Scientific) at a density of 1.5 × 10^5^ cells per well in a total volume of 1 mL DMEM (Thermo Fisher Scientific). Primary and BMI1-transduced bronchial epithelial cells were seeded in 24-well plates at a density of 0.6 × 10^5^ cells per well in a total volume of 1 mL BEGM (Lonza, Slough, UK) media 24 h before transfection. Culture medium was removed from cells and nanocomplex suspension added before centrifugation at 1,500 rpm for 5 min. Cells were incubated at 37°C, 5% CO_2_, and 95% humidity for 4 h, after which OptiMEM was replaced with the complete culture medium. For repeated transfections, the procedure was repeated at 48-h intervals.

### Detection of Cas9-induced genomic editing

Total DNA was extracted using a DNeasy Blood and Tissue Kit (Qiagen, Manchester, UK) and the *CFTR* region of interest in intron 22 amplified by PCR using Q5 Hot Start High-Fidelity DNA Polymerase (New England Biolabs, Hitchin, UK) using appropriate PCR primer sequences (Table S3).

Purified product from PCR reactions with the oligonucleotide primers *CFTR*intron22_F and *CFTR*intron22_R, was subjected to Sanger sequencing (Genewiz, Bishop’s Stortford, UK) to detect indels using ICE software (https://ice.synthego.com/#/; Synthego, Redwood City, CA)^69^ or by TIDE software (http://tide.nki.nl/).^70^ DNA editing efficiency was also assessed by the T7EI assay (New England Biolabs), as described previously^71^ Densitometry was performed of the T7 assay products, and the cutting efficiency of each gRNA was calculated using the following formula: % gene modification = 100 × (1 – (1 – fraction cleaved)^1/2^).

### Off-target indel analysis

CFBE3849 cells carrying the 3849+10kbC>T target mutation were edited four times sequentially as described. Genomic DNA was isolated from edited cells using the DNeasy Blood and Tissue Kit (Qiagen). Predicted off-target sites for U3 and D1 gRNAs were identified using the CCTOP tool (University of Heidelberg, Heidelberg, Germany; https://cctop.cos.uni-heidelberg.de:8043).^72^ Primers for off-target analysis (Table S3) were designed to amplify regions of 250–500 bp surrounding the predicted sites and resulting PCR products were analyzed by Sanger sequencing and quantification of indels by ICE analysis. Further *in silico* analysis of guides U3 and D1 was performed by CRISPOR^73^ and COSMID.^74^

### *CFTR* mRNA transcript analysis

Total RNA was extracted from ALI cultures after 21 days using a RNeasy Mini Kit (Qiagen), resuspended in double-distilled water and reversed transcribed into cDNA using the SuperScript IV Reverse Transcriptase (Invitrogen, Paisley, UK). *CFTR* mRNA and a control mRNA, β-Actin, were quantified by a qRT-PCR assay using Taqman probes (Thermo Fisher Scientific) (Table S4). PCR products of *CFTR* cDNA were amplified with primers *CFTR*ex22_F and *CFTR*ex22_R (Table S3) then analyzed by agarose gel electrophoresis and fragment length analysis (Eurofins Genomics, Wolverhampton, UK). The percentage of the WT fragment was calculated from the area under the curve for each sample relative to the mutant band.

### Immunofluorescent analysis of edited cells

Epithelial cultures were fixed in 4% paraformaldehyde (Merck, Gillingham, UK) for 25 min at room temperature then permeabilized with 0.2% Triton X-100 (Merck) at room temperature for 30 min. Samples were incubated in blocking buffer (3% BSA, 1% fish gelatine, and 0.1% Triton X-100; all from Sigma-Aldrich, Dorset, UK), and 5% donkey serum (Jackson Immunoresearch, Ely, UK) for 2 h at room temperature or overnight at 4°C then primary antibodies to α-tubulin (3 μg/mL), CK8 (4 μg/mL), ZO-1 (3 μg/mL) (all from Abcam, Cambridge, UK) and *CFTR* 596 (Cystic Fibrosis Foundation, Raleigh, NC) (3 μg/mL) were applied and incubated overnight at 4°C. Samples were washed 3× (30 min) with 25% blocking buffer in PBS, pH7.4 (PBS; Thermo Fisher Scientific), followed by staining with secondary antibodies for 1 h at room temperature. After another 3× wash with PBS, samples were nuclear stained with 1 μg/mL 4′,6-diamidino-2-phenylindole (Abcam) for 30 min at room temperature. Images were captured using a Nikon A1R confocal microscope with a ×63/1.3 NA oil immersion lens running Nikon NIS Elements Acquisition Software (Nikon, Amsterdam, the Netherlands).

### Electrophysiological analysis of CFTR function

After 21 days, primary epithelial cells grown at ALI were mounted in modified Ussing chambers (World Precision Instruments, Hitchin, UK) with a solution containing NaCl (117 mM), CaCl_2_ (2.5 mM), KCl (4.7 mM), MgSO_4_ (1.2 mM), NaHCO_3_ (25 mM), KH_2_PO_4_ (1.2 mM), D-glucose (11 mM), and 5 mM HEPES (pH 7.4) at 37°C and bubbled with 21% O_2_ and 5% CO_2_. Measurements were acquired in short-circuit current (Isc) conditions using a DVC-4000 voltage/current clamp (World Precision Instruments) and PowerLab interface (AD Instruments, Oxford, UK). Cultures were equilibrated for a minimum of 20 min prior to the addition of amiloride (10^−4^ M), forskolin (10^−5^ M), CFTR_inh_172 (10^−5^ M), and UTP (10^−4^ M). Drugs were added apically at 5-min intervals with exception of forskolin, which was also added basolaterally. All drugs were obtained from Sigma-Aldrich, Dorset, UK. Data were collected using LabChart (version 7) software (AD Instruments).

### Statistical analysis

The significant differences between 2 groups were calculated using a Student’s t-Test. When more than 2 groups were being compared, one-way ANOVA was used. Any p values of less than 0.05 were marked with ∗. Data was analyzed using GraphPad Prism version 7.0 and expressed as mean ± SD.

## Data and code availability

All data are available on request to Stephen Hart (s.hart@ucl.ac.uk).

## Supplemental information

Document S1. Figures S1–S4 and Tables S1–S4

Data S1. U3 CRISPOR

Data S2. D1 CRISPOR

Data S3. U3 COSMID

Data S4. D1 COSMID

Document S2. Article plus supplemental information
