# Supplementary material for: Molecular and functional correction of a deep intronic splicing mutation in CFTR by CRISPR-Cas9 gene editing
Source: Mol Ther Methods Clin Dev. 2023 Oct 18;31:101140. doi: 10.1016/j.omtm.2023.101140 (PMC10661860; doi:10.1016/j.omtm.2023.101140)
Supplement: Document S1. Figures S1–S4 and Tables S1–S4 [file mmc1.pdf]

OMTM, Volume *31*

Supplemental information

Molecular and functional correction of a deep intronic splicing mutation in *CFTR* by CRISPR-Cas9 gene editing

Amy J. Walker, Carina Graham, Miriam Greenwood, Maximillian Woodall, Ruhina Maeshima, Michelle O'Hara-Wright, David J. Sanz, Ileana Guerrini, Ahmad M. Aldossary, Christopher O'Callaghan, Deborah L. Baines, Patrick T. Harrison, and Stephen L. Hart

Supplemental Figures and Data

Table S1 Cytotoxicity of RTN RNP transfections NHBE *BMI1*-GFP cells (n=3 per group) were transfected with RTN formulations (RNP: DOTMA/DOPE (L): Peptide (P)) that target GFP then viability analysed at 24h or 48h by ToPro3 viability assays. There was no reduction in viability at either time point compared to untransfected cells. Mean values were compared by a Student t test.

Group	Live cells %	SD	*p*
untransfected	98.10	0.44	
24h	98.03	0.34	0.85
48h	99.42	0.48	0.02

Data files: Off -Target Analysis by CRISPOR and COSMID

U3 and D1 guide RNA sequences were analysed by a) CRISPOR and b) COSMID. The two target sequences were entered into the CRISPOR web browser (http://crispor.tefor.net/crispor.py), and the off-target files exported as Excel files, then ranked by CFD scores. Sites with a CFD <0.02 were excluded. The five sites for U3 and D1 selected for analysis using CCtop for each gRNA are highlighted.

a) U3 gRNA CRISPOR.xls

b) D1 gRNA CRISPOR.xls

c) U3 gRNA COSMID.xls

d) D1 gRNA COSMID.xls

Table S2: Guide RNA sequences

gRNA name	Spacer sequence (5'-3')	PAM	sense
Upstream 1 (U1)	CATTTTAATACTGCAACAGA	TGG	-
Upstream 3 (U3)	CTTGATTTCTGGAGACCACA	AGG	+
Downstream 1 (D1)	TTGATCCAACATTCTCAGGG	AGG	+

Table S3: PCR primer sequences

Name	Sequence 5' – 3'	Product size
CFTRintron22_F	TCCTCTCAAAATGCCTACTGG	993 bp
CFTRintron22_R	GGTTGGGAAAGACTGGATGA	
CFTRex22_F	5'-GATCTGTGAGCCGAGTCTT	465 bp
CFTRex24_R	GATCACTCCACTGTTCATAGG	
CFTRin22_F	ATCATATCAGCACAAACACC	742 bp
CFTRin22_R	GGGAAAGACTGGATGAAGA	

Table S4: Taqman Assays (FAM-labelled probes)

Name	Assay ID	Supplier
Human ACTB	Hs01060665_g1	Thermo Fisher Scientific
Human CFTR	Hs00357011_m1	Thermo Fisher Scientific

a

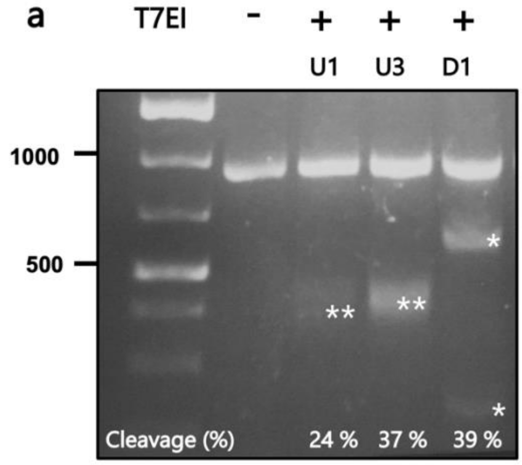

b

gRNA	Cleavage site	Fragment size 5'	Fragment size 3'
U1	522	522	471
U3	484	484	509
D1	663	663	330

c

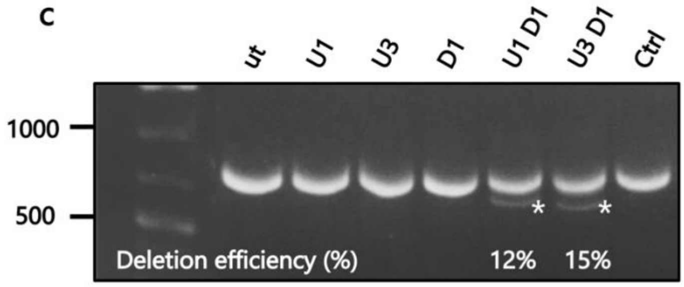

d

gRNA pairs	Deletion product size (bp)
U1 D1	141
U3 D1	179

Figure S1. gRNA validation in HEK293T cells. **a** T7 Endonuclease I (T7EI) mismatch assay products from CFTR intron 22 in HEK293 cells transfected with Cas9 RNPs with either upstream (U1, U3) or downstream (D1) gRNAs, using Lipofectamine® 2000. Cleaved product bands, indicated with an asterisk, were quantified by densitometry. Interestingly T7 cleavage products were smaller than expected in the Table **b** with guides U1 and U3 which we cannot explain at present, although it is clear that cleavage has occurred. **b** Predicted gRNA-guided Cas9 cleavage site and resulting fragment sizes**.** **c** Targeted excision in CFTR intron 22 induced by pairs of gRNA-Cas9. HEK293T cells were transfected with Cas9 and individual or paired, upstream (U1, U3) and downstream (D1) gRNAs in RNP format using Lipofectamine 2000. Lower molecular weight species represent products of targeted excision DNA band quantified by ImageJ. Deletion efficiency

is quantified as a % cleaved product of the total product. **d** Expected deletion size induced by paired gRNAs.

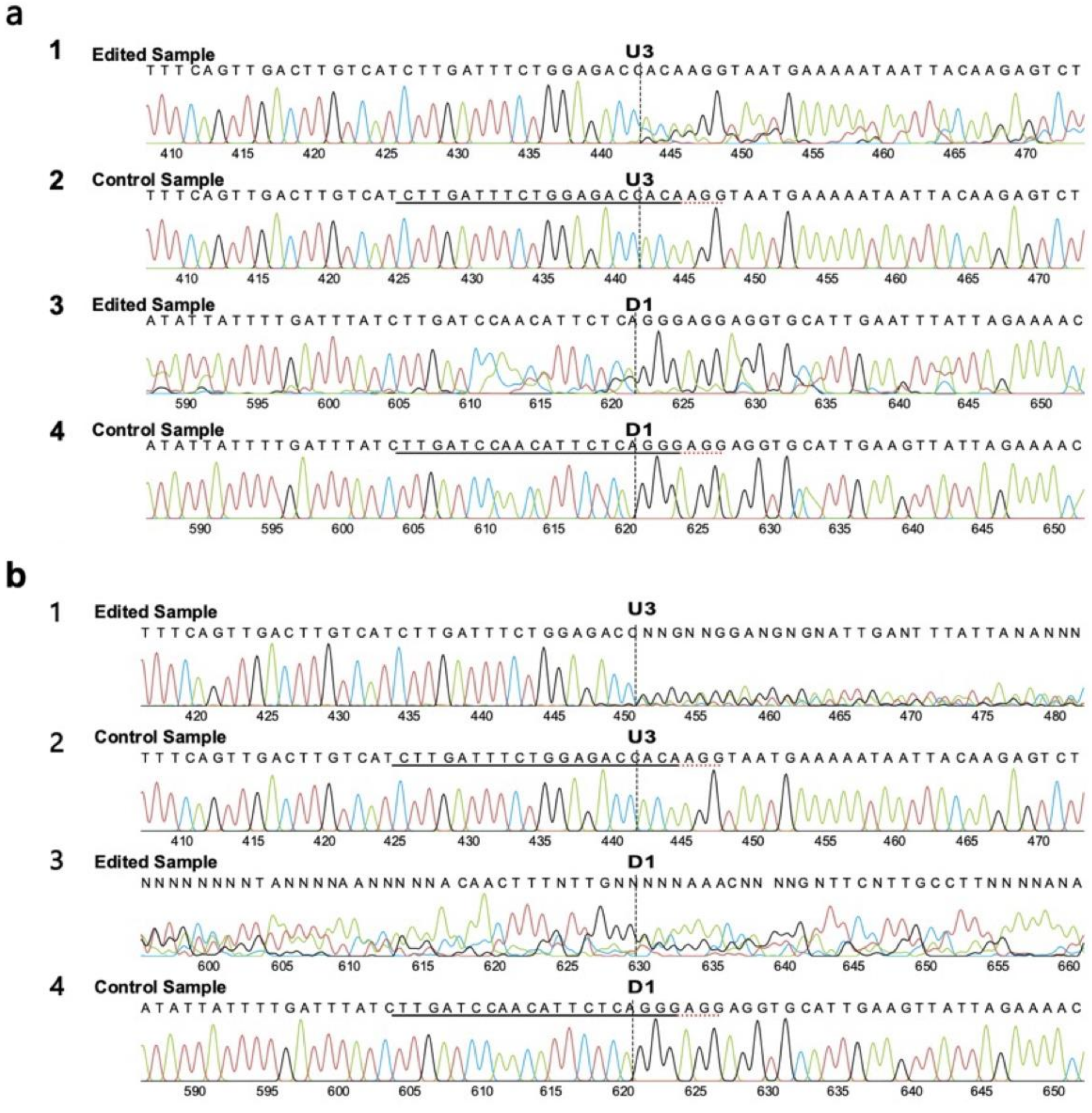

Figure S2. Sanger sequence analysis of edited CFBE 3849+10kb C>T cells. Cells were transfected once with U3D1 gRNAs in RNP complexes using nanoparticles **a**, or by four sequential transfections **b**, before genomic DNA extraction and PCR amplification of the region of interest. Amplicons were Sanger sequenced and analysed using ICE software. Dotted lines indicate

the gRNA cutting sites. A heterogeneous base population at the cut sites in edited samples (**1,3**) indicates indel formation due to NHEJ repair and is more evident after four transfections. PCR amplification of control samples exhibited a clean sequence (**2,4**).

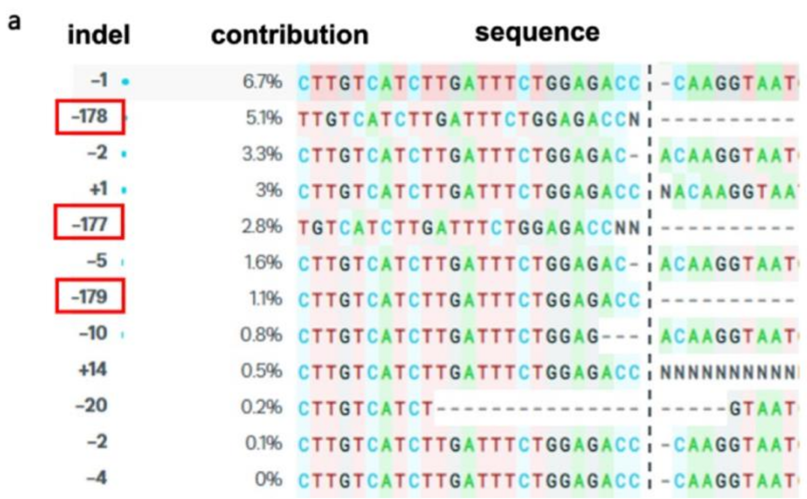

b

x1 round transfection	
Targeted excision (%)	9
Indels (%)	26
Pearson's regression co-efficient (r^2)	0.94

Figure S3. Inference of CRISPR Edits (ICE) analysis of CFBE3849+10kb C>T cells. a CFBE3849+10kb C>T cells transfected with nanoparticles containing Cas9 +U3D1 RNP were analysed for editing in CFTR intron 22 by SYNTHEGO ICE. Sequences highlighted in red boxes show a deletion of ~178 bp, indicative of successful cutting by both gRNAs upstream and downstream of mutation. **b** The targeted excision rate was 9%, with 26% total indels, and an r^2 value of 0.94.

Rep1

a

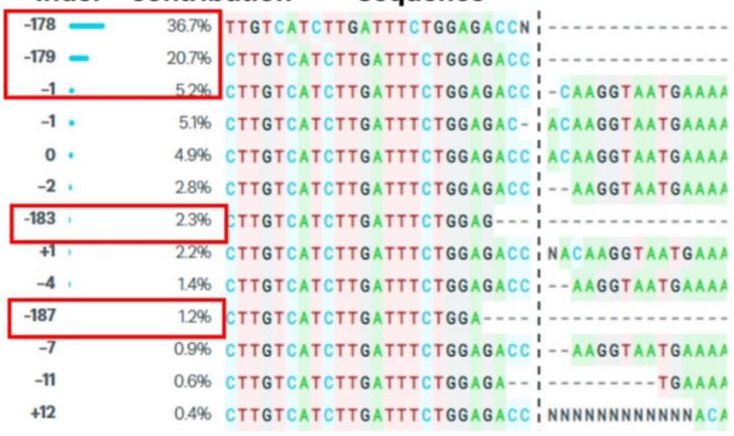

b

x4 rounds transfection	
Targeted excision (%)	60.9
Indels (%)	83
Pearson's regression co-efficient (r^2)	0.88

Rep 2:

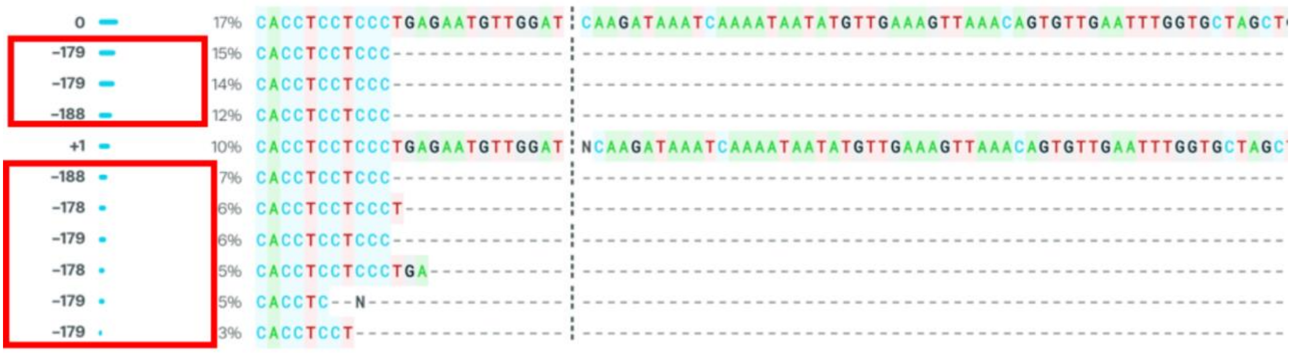

Rep 3

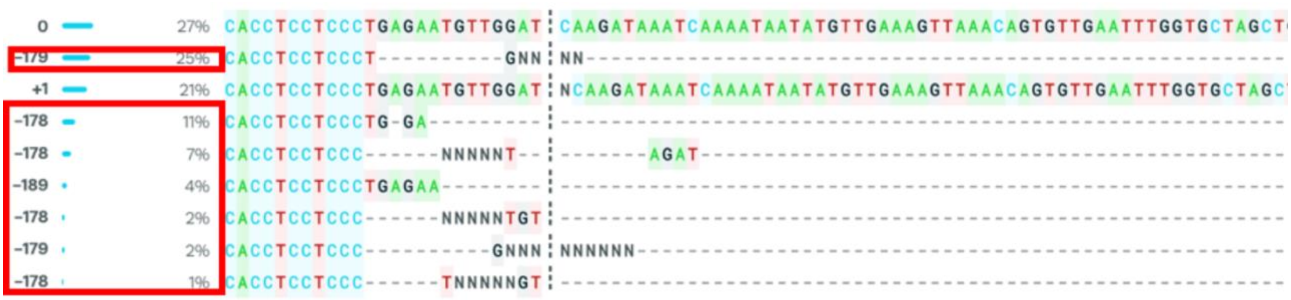

Figure S4. Inference of CRISPR Edits (ICE) analysis of CFBE3849+10kb C>T cells. a CFBE3849+10kb C>T cells were sequentially transfected four times with nanoparticles containing Cas9 +U3D1 RNP then analysed for editing in CFTR intron 22 by SYNTHEGO ICE)(n=3).

Sequences highlighted in red boxes show a deletion of 178-188 bp, indicative of successful cutting by both gRNAs upstream and downstream of mutation.
